# Professional or Unprofessional? That Is the Question! Mr. Wakley's Report of the Late Trial

**Published:** 1829-04-01

**Authors:** 


### XVIII.

### PROFESSIONAL OR UNPROFESSIONAL? THAT IS THE QUESTION!

[Mr. Wakley's Report of the Trial.]

Mr. Lambert has been turned out of two Societies for an "UNPROFESSIONAL REPORT," and we are now to call the attention of our surgical brethren to a REPORT by Mr. Lambert's Master—the "REPORT of the TRIAL." Mr. Wakley is constantly calling upon the SURGEONS, and especially the GENERAL PRACTITIONERS of England, to rally round his standard, as their leader and representative in the "ENSUING AUTUMN." Now we request that they will carefully inspect the FRONTISPIECE and plates of the REPORT in question, for there they will see, *veluti in speculo*, an emanation from the great mind of their self-appointed leader and teacher. A human figure (which Wakley could never have seen) with agonised features and manacled limbs—with a gash, (or rather gulf) reaching from near the pubes to the os coccygis, and measuring in length the distance between the sternum and chin, while the neck is on the stretch!!—such a figure is presented to the SURGEONS and GENERAL PRACTITIONERS of England, to teach them how to operate for lithotomy!! In compliment to the learning of his surgical readers, Mr. Wakley has prefixed a glossary to his work, in which, among many other highly important pieces of intelligence, he gives them to know that—the "abdomen" means the "belly"—that calculus signifies "the stone *found* in the bladder;" (of course it is not a calculus till it is *found* there)—that integuments mean skin—that the subclavian artery is "a vessel which passes under the collar-bone"—that the "urethra is the canal through which the urine *escapes* from the bladder"—and to crown this monument of surgical literature, that "the umbilicus is the navel." Mr. Wakley seems to have been taken with a fit of unbounded generosity towards his surgical readers. He has gone to the expense of lithographing a whole ARMAMENTUM LITHOTOMICUM, by means of which the country-surgeons will be able to learn the names of nine instruments employed in cutting for the stone. To each figure the name of the instrument is affixed, and in order to impress these names on his readers' memories, they are again given in a distinct list. Now, if this profusion of information poured out upon the surgical profession for the trifling sum of *four shillings*, does not bind them in chains of gratitude to the "HERO of REFORM," we know not what will!

But we must be serious upon this point. We appeal to every surgeon in England, who takes the trouble to examine Mr. Wakley's book, whether it is not even more "UNPROFESSIONAL" than the "Report" for which Lambert has been degraded. It is evidently designed, not to convey any surgical information to his brethren; but to create a horror of surgery among the vulgar. To exhibit a cast of the unfortunate patient in a Court of Justice was bad enough. To expose such a figure as that which graces the Report, to the idle gaze of the millions who pass along the Strand, is the most disgraceful act (professionally speaking) that ever surgeon did! We ask Mr. Wakley—we ask his medical brethren, if this be an honourable, manly, and surgeon-like mode of vindicating his own conduct, and impugning that of Mr. Cooper,—or is it a mean, dastardly, vindictive, and coarse device, calculated to excite the angry feelings of the ignorant mob against a brother-practitioner? Let Mr. W.—let the profession itself answer these questions!! Human ingenuity never devised a procedure better adapted to engender disgust against the whole profession of surgery, and horrify the non-professional public, than that which is adopted in this REPORT, and practically enforced at 210, in the Strand. If Mr. Wakley or his adherents believe that they can throw odium on *part* of the profession, without injuring the *whole*, they allow their passions to obscure their reason. If a publication were to start in the law, for the purpose of representing the judges and barristers as a set of knaves, and argued that, by so doing, it would induce a belief that all attornies were honest men, what would be thought of the intellects of the editor? Yet such precisely are the tactics of Mr. Wakley and his associates. By representing HOSPITAL SURGEONS as ignorant and clumsy, the public, he tells us, must naturally draw the inference that *all other surgeons* are learned and dexterous—Q. E. D!! If the Brodies, the Guthries, the Bells, the Coopers, the Travers; and the Stanleys, who have a wide range of hospital experience for their assistance, be incapable of conducting capital operations, it follows, from Mr. Wakley's logic, that the Lamberts, the Lees, the Claphams, and the Gilberts, are the men to whom the public are to look for high operative surgery! Would any other than Wakley draw this conclusion, unless he were an inmate of Bedlam, or had recently made his escape thence?

If Mr. Wakley were as busily engaged in practising his profession, as in traducing its most distinguished members, he would long since have learnt that the greatest difficulty which medical men encounter in their intercourse with the public, is this very distrust (unjust and ungenerous as it is) of their exertions, which he labours, day and night, to augment. Practitioners know, to their sorrow, that whenever a case goes not on smoothly, quickly, and favourably, (cito, tute, ac jucunde,) the patient begins to suspect the skill of the medical attendant; and, not unfrequently casts reflections on his professional character! We ask the surgeon or the physician (for we need not ask Mr. Wakley, who seems to know or care nothing about the matter) whether this distrust of the private practitioner's knowledge is to be lessened by representing almost the whole of the heads of the profession as stupid, ignorant, and knavish? The answer is obvious. The distrust would be increased tenfold, if such a preposterous belief could be generally infused into the minds of the public. We fearlessly maintain, then, that Mr. Wakley's writings are not merely *unprofessional*, but that they are ANTI-PROFESSIONAL, and tend, in *every way*, to depress the whole medical character of the country. As to the FRONTISPIECE to the "REPORT of the TRIAL," we solemnly and publicly denounce it as a disgrace to the age in which we live—as a stigma on the science we profess—as a blot on the surgical annals of the country which gave it birth! The frontispiece in, and the canvas placard over, the shop-window of Mr. Wakley, may make the ignorant stare, but cannot fail to make the sensible grieve! They afford, at once, the signal and the proof that he has ceased to appeal to the PROFESSION—not indeed before it has begun to turn a deaf ear to him—and has addressed himself to the mob, whose passions and prejudices he may yet turn to some account. His lithographs and paragraphs may pass among the vulgar for SURGERY, but they must be considered as VULGARITY among surgeons.

